# The prospective outcome of the monkeypox outbreak in 2022 and characterization of monkeypox disease immunobiology

**DOI:** 10.3389/fcimb.2023.1196699

**Published:** 2023-07-18

**Authors:** Muhammad Suhaib Qudus, Xianghua Cui, Mingfu Tian, Uzair Afaq, Muhammad Sajid, Sonia Qureshi, Siyu Liu, June Ma, Guolei Wang, Muhammad Faraz, Haleema Sadia, Kailang Wu, Chengliang Zhu

**Affiliations:** ^1^ Department of Clinical Laboratory, Institute of Translational Medicine, Renmin Hospital of Wuhan University, Wuhan, Hubei, China; ^2^ State Key Laboratory of Virology, College of Life Sciences, Wuhan University, Wuhan, China; ^3^ RNA Therapeutics Institute, Chan Medical School, University of Massachusetts Worcester, Worcester, MA, United States; ^4^ Krembil Research Institute, University of Health Network, Toronto, ON, Canada; ^5^ Department of Pharmacy, University of Peshawar, Peshawar, Pakistan; ^6^ Department of Microbiology, Quaid-I- Azam University, Islamabad, Pakistan; ^7^ Department of Biotechnology, Baluchistan University of Information Technology, Engineering and Management Sciences (BUITEMS), Quetta, Pakistan

**Keywords:** monkeypox, MPOX infection, MPOX vaccination, MPOX immunity, MPOX diagnosis

## Abstract

A new threat to global health re-emerged with monkeypox’s advent in early 2022. As of November 10, 2022, nearly 80,000 confirmed cases had been reported worldwide, with most of them coming from places where the disease is not common. There were 53 fatalities, with 40 occurring in areas that had never before recorded monkeypox and the remaining 13 appearing in the regions that had previously reported the disease. Preliminary genetic data suggest that the 2022 monkeypox virus is part of the West African clade; the virus can be transmitted from person to person through direct interaction with lesions during sexual activity. It is still unknown if monkeypox can be transmitted via sexual contact or, more particularly, through infected body fluids. This most recent epidemic’s reservoir host, or principal carrier, is still a mystery. Rodents found in Africa can be the possible intermediate host. Instead, the CDC has confirmed that there are currently no particular treatments for monkeypox virus infection in 2022; however, antivirals already in the market that are successful against smallpox may mitigate the spread of monkeypox. To protect against the disease, the JYNNEOS (Imvamune or Imvanex) smallpox vaccine can be given. The spread of monkeypox can be slowed through measures such as post-exposure immunization, contact tracing, and improved case diagnosis and isolation. Final Thoughts: The latest monkeypox epidemic is a new hazard during the COVID-19 epidemic. The prevailing condition of the monkeypox epidemic along with coinfection with COVID-19 could pose a serious condition for clinicians that could lead to the global epidemic community in the form of coinfection.

## 1 Introduction

The monkeypox virus (MPOX) is an enveloped double-stranded DNA orthopoxvirus belonging to the Poxviridae family that causes a zoonotic viral illness in humans (McCollum and Damon, 2014). The name “monkeypox” probably derives from the fact that MPOX was first identified in 1958 in Singaporean research monkeys (Cynomolgus) transported to the Statens Serum Institute in Copenhagen, Denmark (Shafaati and Zandi, 2022; Zandi et al., 2023). However, mice and other small animals seem more likely to be natural hosts for MPOX (Cho and Wenner, 1973; Cohen, 2022). The deadly smallpox virus, variola virus (VARV), is a member of the Orthopoxvirus genus. Like smallpox, monkeypox can cause similar symptoms in humans but has a lower death rate (Ježek et al., 1987; McCollum and Damon, 2014).

There are two distinct clades of the MPOX, one originating in the Central African (Congo Basin) region and the other in Western Africa. The Cameroon is the only country where both viral clades are found, marking a geographic split between the two groups. In addition to the Cameroon, Nigeria, Liberia, Ivory Coast, Sierra Leone, and the United States have all had epidemics of the West African clade (imported from Ghana). Gabon, Cameroon, the Republic of the Congo, the Central African Republic (CAR), Sudan, and the Democratic Republic of the Congo (DRC) have all been identified as home to the Central African clade (Ježek et al., 1987; Chen et al., 2005; Likos et al., 2005; Sbrana et al., 2007). These two clades are also very different in terms of epidemiologic and clinical characteristics, on top of the apparent disparities in geography. More than 2000 cases are recorded annually from the Central African clade, which is endemic to the Democratic Republic of the Congo, than from the West African clade, found primarily in Nigeria (Mwamba et al., 2014). The Central African strain may cause more severe symptoms and have more potential for transmission than the West African clade. There has never been evidence of human-to-human transmission, and the case fatality rate (CFR) for the West African clade is relatively low (1%). On the other hand, the CFR of the Congo Basin clade may reach as high as 11%, and up to six independent transmissions between humans have been detected (Ježek et al., 1987). Another assessment identified a similar disparity in CFR between the Central African clade (10.6%; 95% CI: 8.4%-13.3%) and the West African clade (3.6%; 95% CI: 1.7%-6.8%) (Bunge et al., 2022).

This article discusses the specific pathological and epidemiological aspects of MPOX, as well as the propagation and immunopathogenesis of this reemerging outburst. Compared with other poxviruses, we investigate the potential host defense mechanisms against MPOX. Moreover, preventative strategies like Vaccinations, therapies, and information shortages are also discussed.

### 1.1 Search strategy

The keywords “Orthopoxvirus, Monkeypox virus, MPOX Central African Clade, Immunopathogenesis of MPOX, were used with Boolean combinations. The literature search and relevant evaluation were conducted using PubMed, Web of Science, Global Health, WHO, and Google Scholar databases. Articles found were considered potential referral sources. We also looked for other poxvirus, like Vaccinia virus and Variola virus, to draw a comparative analysis. The searches were performed up to early November 2022.

## 2 Epidemiology

Human cases of MPOX were first reported in the 1970s in several African countries, but the virus has spread more rapidly over the past 20 years (Heymann et al., 1998). The Democratic Republic of the Congo (DRC), Côte d’Ivoire, Sierra Leone, Liberia, Nigeria, and Cameroon were among the six African countries where 48 confirmed and suspected cases were reported (Foster et al., 1972; Breman et al., 1977; Breman et al., 1980; Bunge et al., 2022). MPOX was confined to Africa between 1980 and 2000, with no other infections or transmissions reported. There has been a rise in the number of reported cases of MPOX throughout Africa, The Democratic Republic of the Congo (DRC) being the epicenter (with over 800 confirmed and suspected cases) and occasional reports coming in from other countries like the CAR, Gabon, Côte d’Ivoire, and Cameroon (Merouze and Lesoin, 1983; Khodakevich et al., 1985; Jezek et al., 1986; Ježek et al., 1988; Meyer et al., 1991; Tchokoteu et al., 1991; Aplogan and Szczeniowski, 1997; Bunge et al., 2022). MPOX cases were reported in the CAR, Liberia, Nigeria, South Sudan, Sierra Leone, Cameroon, and the DRC between 2000 and 2020 (Foster et al., 1972; Tchokoteu et al., 1991; Learned et al., 2005; Formenty et al., 2010; Berthet et al., 2011; Reynolds et al., 2013; McCollum and Damon, 2014; Doshi et al., 2019; Reynolds et al., 2019; Bunge et al., 2022). The DRC reported over 20,000 suspected cases during the same period, while Nigeria reported 181 cases. In 2003, 47 confirmed and suspected cases of MPOX infection were reported in the United States. Initially, imported Gambian pouched rats infected prairie dogs, which then spread the disease to humans (Huhn et al., 2005; Reynolds et al., 2007; Yinka-Ogunleye et al., 2019; Bunge et al., 2022). MPOX infections have been reported in Israel (2018), the UK (2018-2021; 7 cases), Singapore (2019), and the US (2021; two cases) (Huhn et al., 2005; Vaughan et al., 2018; Erez et al., 2019; Vaughan et al., 2020; Yong et al., 2020; Hobson et al., 2021; Adler et al., 2022; Bunge et al., 2022; Costello et al., 2022; Rao et al., 2022).

### 2.1 Re-emergence of MPOX outbreak in the world

**Figure 1:**
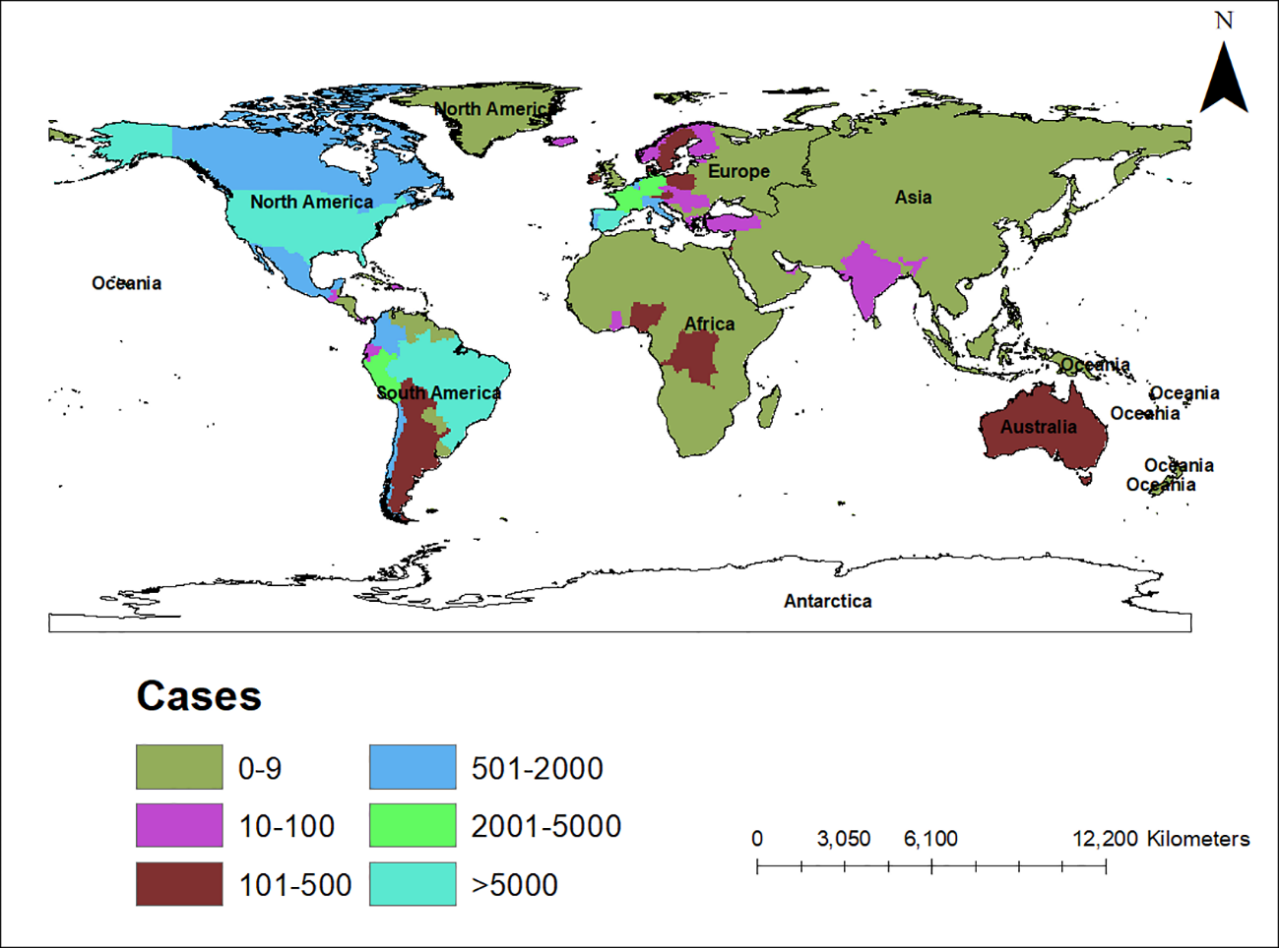
Map showing the spread of the monkeypox epidemic around the world from May to November 2022, including confirmed and suspected cases. The data shown here are as of November 10, 2022, and were retrieved from Global Health (https://map.monkeypox.global.health/country). This diagram was created using Data wrapper.

**Figure 2:**
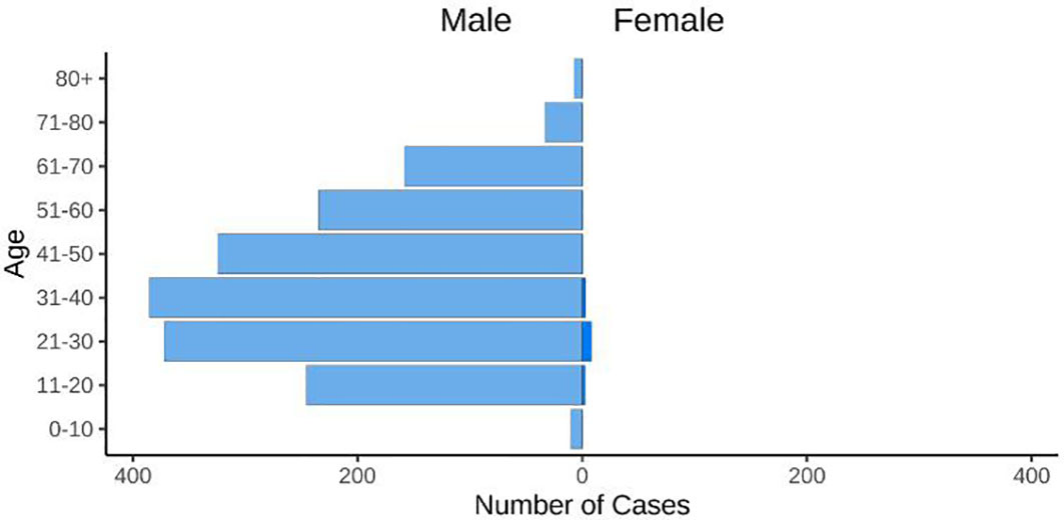
The figure displays the age and gender distribution. 98% of cases were typically diagnosed in people between the ages of 21 and 50 (https://map.monkeypox.global.health/country).

An alarming rise of MPOX cases has been recorded across the globe, including in Australia, the Middle East, the Americas, and Europe, since May 2022 (Kraemer et al., 2022; Minhaj et al., 2022; Otu et al., 2022; Shafaati and Zandi, 2023) Globally, as of November 10, 2022, nearly 79,500 confirmed and 1,300 probable cases were recorded in global health database (Supplementary Data) (https://www.cdc.gov/poxvirus/monkeypox/response/2022/worldmap.html; Kraemer et al., 2022), and 53 deaths out of which 40 deaths were reported with no history of monkeypox cases while other 13 were reported in locations that have historically reported monkeypox (https://map.monkeypox.global.health/country) (**Figure 1**). Monkeypox has re-emerged in Singapore for the first time since 2019, with multiple imported and local cases recently confirmed (Yong et al., 2020; Channel News Asia, 2022). The World Health Organization (WHO) proclaimed a worldwide health emergency due to the spread of monkeypox on July 23, 2022 (Kimball, 2022). The decline in smallpox immunity and the end of smallpox vaccinations are possible contributors to the present epidemic (Reynolds and Damon, 2012). Vaccination against smallpox has also been shown to prevent monkeypox. According to a study conducted in Zaire in 1988, smallpox immunization (during a nationwide smallpox vaccination program commencing 12 years prior to data collection) reduced the risk of monkeypox infection by about 85% (Fine et al., 1988). Another study indicated that patients vaccinated against smallpox were less likely to experience serious symptoms and long-term (39.5 vs 74%) consequences from MPOX infection (Ježek et al., 1987). A newly published global research of 528 MPOX infections (527 men and 1 woman) identified from 27 February 2022 to 24 June 2022, across 16 nations and 5 continents. The median age of patients was 38 y (range 18–68 y), which includes 98% of the people with infection who were gay or bisexual. The most recent epidemics have concentrated men who engage in sexual activity with other men (MSM) (**Figure 2**) (Otu et al., 2022; Thornhill et al., 2022).

#### 2.1.1 Genome and phylogeny of MPOX

The genetic mutations of the monkeypox virus (MPOX) are still a mystery, making it difficult to determine if they are responsible for the latest outbreaks. The 2022 epidemic can be traced back to a single subclade, specifically B.1 lineage within the West African clade (MPOX 3^rd^ Clade) (Isidro et al., 2022). 46 single-nucleotide polymorphisms (SNPs), including 24 non-synonymous ones, have been identified as unique to this subclade (Luna et al., 2022). Recent molecular epidemiology investigations have observed more genomic variation than expected among the outbreak sequences, which may indicate rapid evolution and APOBEC3 editing (Gigante et al., 2022; Luna et al., 2022). However, whether these genetic variations are responsible for the observed epidemiological phenotype is unclear. The capacity of the virus to disseminate is thought to be influenced by 3-non-synonymous single-nucleotide polymorphisms (P722S, M1741I, and D209N) in the primary antibody target, surface glycoprotein B21R (Hammarlund et al., 2005; Luna et al., 2022). MPOX, on the other hand, is an intricate virus that has a DNA-based genome that is almost six times bigger (197 kb) than the RNA-based genome that SARS-CoV-2 possesses. Computational analyses have revealed that the MPOX Zaire strain, prevalent in Central Africa, had at least 190 open reading frames (ORFs). The core section of the MPOX genome contains genes known to have a crucial role for orthopoxviruses (between ORFs C10L and A25R) (Shchelkunov et al., 2001; Shchelkunov et al., 2002; Kugelman et al., 2014). However, compared to the genomes of other orthopoxviruses, a portion of ORFs in the MPOX genome are either absent or shortened. As a result, determining the possible influence of certain genetic changes on the viral phenotype is complicated and laborious. However, in contrast to RNA-based viruses, MPOX’s DNA-based genome has a considerably higher potential to correct errors generated during viral replication (Sanjuán and Domingo-Calap, 2016). The West African clade is less virulent than the Central African clade, and this may be due to the disruption of multiple ORFs encoding immune circumventing genes (Shchelkunov et al., 2001; Chen et al., 2005; Weaver and Isaacs, 2008; Reynolds and Damon, 2012). The virus can infect cells in two different ways, i.e. as an intracellular mature virus and the extracellular enveloped virus, which differ in their surface glycoproteins and different infection mechanisms. Although MPOX duplication is complicated, it is widely believed to be the same as other orthopoxviruses (McFadden, 2005). There have been many suggestions as to which surface receptors, such as heparan sulfate and chondroitin sulfate, are used by MPOX to gain entry, but none have been confirmed as of yet. Virus-host interactions in the vaccinia virus (VACV) have been linked to the surface proteins H3, A27, and D8 (Chung et al., 1998; Hsiao et al., 1999; Lin et al., 2000). As a result of its interaction, VACV forms a complex of 11 conserved proteins called the entrance fusion complex, which allows it to enter the cell (Moss, 2016). Genetic accordions (multistep operates in various gene mutations and applications that permit poxviruses to circumvent host antiviral defenses) are one-way VACV might alter phenotypes.). To counteract the host’s kinase protein R (PKR)-mediated defenses, the K3L gene is highly elevated in the VACV variant. This, in combination with a helpful point mutation in a similar gene, allows the virus to infect and replicate in humans successfully (Elde et al., 2012). Genetic accordions like these have not been shown in MPOX, but it is important to investigate if they might be a credible tool for the evolution and transmission of this virus.

#### 2.1.2 Novel viral mutations and viral strains responsible for the 2022 outbreak

DNA viruses like MPOX exhibit fewer mutations than RNA viruses, like HIV and SARS-CoV-2 (Sanjuán and Domingo-Calap, 2016). According to reports, the MPOX virus detected from the outbreak in 2022 appears to contain more mutations. Surprisingly, the 2022 isolates shared 40 mutations that set them apart from their nearest variant. A virus-like MPOX would be expected to accumulate numerous mutations throughout conventional evolutionary timescales, which may take more than 50 years. Possible transmission-improving mutations in the MPOX are under investigation. Although the MPOX has a slower mutation rate than other DNA viruses, it is still possible that adaptive mutations of the MPOX will accumulate in response to a particular selection pressure (Zhang et al., 2022). Some mutations have no negative consequences on the virus, while others can be damaging and even exploit weaker strains. There is a lack of data on MPOX’s interactions with the host or the effects of these alterations on viral replication rates. Enzymes in the immune systems of some hosts (humans) have been shown to cause mutations in viruses (Siggs, 2014). In 2017, MPOX virus evolution appeared to accelerate, based on available sequences that have been circulating in humans, which indicated a mutation rate for this virus that is roughly 10 times greater than the normal mutation rate. There is a paucity of knowledge on the effects of specific mutations or how they interact with the host (human) in newly discovered MPOX strains. Researchers are concerned by many MPOX gene sequence variants in the present outbreak; more work is required to determine how these mutations contribute to disease transmission (Kumar et al., 2022).

On June 3, 2022, the Centers for Disease Control and Prevention in Atlanta, Georgia, released a paper indicating that there are likely two different clades of MPOX responsible for the outbreaks outside of Africa. The Centers for Disease Control and Prevention sequenced 10 viral isolates from recent outbreaks in the United States and determined that these isolates are distinct from viruses sequenced by many countries implicated in the widespread outbreak that originated in Europe and is currently spreading throughout the world. The European outbreak appears to be fueled mostly by the homosexual, bisexual, and MSM communities. Three of the ten US isolates seemed to be genetically unique from the other seven. Although the three distinct isolates appear to share a common ancestry, they also appear to be distinct from one another. It’s fascinating to note that these 3 unique isolates appear to have spread from different parts of the world. A preliminary investigation suggests that one started in Nigeria, another in West Africa, and a third in the Middle East or East Africa (Branswell, 2022; Kumar et al., 2022).

## 3 Clinical features and transmission of MPOX

MPOX infection typically causes a high temperature (38.5 to 40.5 degrees Celsius), headache, and muscle pain between 5 and 21 days after exposure. Enlargement in the inguinal cervical or maxillary lymph nodes (lymphadenopathy) may suggest MPOX infection which then needs diagnostic confirmation (Ježek et al., 1987; McCollum and Damon, 2014). Researchers in Portugal observed that during the outbreak, groin lymphadenopathy was more prevalent than cervical and axillary lymphadenopathy. After the fever develops, the rash appears on the face, mouth (enanthem), and tongue, which then spreads to the rest of the body. As the disease progresses, lesions in the mouth can make it difficult to eat and swallow (Ježek et al., 1987; Duque et al., 2022). Yet, in the current epidemics, several unusual cases have been reported. Patients with MSM have been observed to suffer from genital lesions that spread to other body parts, anal ulcers, and possibly a more localized distribution of skin lesions documented in earlier outbreaks (Antinori et al., 2022; Duque et al., 2022; Hammerschlag et al., 2022).

The number of lesions is a useful measure of disease severity because of higher lesion count indicates a direr prognosis. Ocular infections can cause irreversible blindness, and patients may also develop respiratory and gastrointestinal problems. Dermal bacterial infections are more common in people with skin lesions, especially those who haven’t been immunized against smallpox (Ježek et al., 1987; Huhn et al., 2005; Learned et al., 2005; Adler et al., 2022). A lesion normally goes through four stages before it scabs and falls off permanently: macular, papular, vesicular, and pustular. The patient is usually no longer infectious when the lesions have crusted up. Despite this, it has been reported that scabs still retain detectable amounts of MPOX DNA, suggesting the existence of infectious viral material even after the scab has fallen off. Interestingly, smallpox scabs have been found to contain a live variola virus (VARV) (Downie and Dumbell, 1947; McCollum and Damon, 2014; Pittman et al., 2022).

To this day, MPOX’s reservoir host is a mystery. However, rodents native to Africa have been implicated as possible disease vectors. MPOX transmission occurs through contact with skin lesions, bodily fluids, or respiratory droplets from affected animals (Angelo et al., 2019). This virus infects hosts when it enters the body through weakened mucous membranes, weakened respiratory systems, or broken skin (eyes, nose, or mouth). Scratches, bites, preparing bush meat, or contacting animal body fluids or lesion material are all possible transmission routes from animals to humans (Grant et al., 2020). Large respiratory droplets from sneezing, coughing, etc., can spread an infection from person to person. Due to the limited range of respiratory droplets (just a few feet), prolonged face-to-face contact is required for transmission. Contact with the viral lesion or body fluids, as well as indirect contact with infected objects like clothing or infected linens, are other ways that the virus can spread from person to person.

MPOX can be transmitted from mother to child by vertical transmission during pregnancy. Only one of four pregnant women in the DRC contracted MPOX and delivered a healthy baby. In the first trimester, two women had miscarriages, and one had a stillbirth. A stillborn fetus’s autopsy revealed fetal edema, noticeable hepatomegaly, and peritoneal effusion in addition to diffuse patchy skin lesions affecting the head, trunk, and extremities (including the palms and soles). In another study, lesion formation on the maternal placental surface and fetal death were reported in 80% of MPOX-infected women in the DRC. Although considering the location of the investigations, these patients were likely infected with the Central African clade of MPOX; this information was not included in the research (Mbala et al., 2017; Pittman et al., 2022). According to studies done in Zaire between 1980 and 1985, the death rate from MPOX infection was 14.9 percentage points greater in children under the age of 4 compared to those over the age of 10. Possible explanations for this discrepancy include different immunological responses (Ježek et al., 1987; Carvajal and Vigil-De Gracia, 2022; Centers for Disease Control and Prevention, 2022; Vouga et al., 2022). Given the severity of monkeypox in infants and toddlers, future public health actions to control MPOX and limit the probability of adversative consequences are critical.

Recently, MPOX was found in the semen of three Italian men, lending credence to the hypothesis that this virus might be transmitted sexually (Alakunle et al., 2020; Antinori et al., 2022). Exclusive genital lesions caused by MPOX have been reported, which may be evidence of the virus’s preferential tropism in the testes. The testes may serve as a reservoir for MPOX since they are an immune-privileged tissue, but this hypothesis needs to be explored further (Davido et al., 2022; Forrester et al., 2022). However, recent animal studies reveal that the related VACV favors male genitalia and ovarian organs (tropism) (Zhao et al., 2011; Prow et al., 2018). Transmission of MPOX may be facilitated by contact with the rectal mucosa, as viral shedding has been described in feces (Rimoin et al., 2010). Viruses like human immunodeficiency virus type 1 (HIV-1) have persisted in these tissues (Khoury et al., 2017). An increased presence of immunological events revealing mucosal injuries was found in the rectal mucosa of MSM, compared to those of heterosexuals, in a recent study (Kelley et al., 2017). Infectious pathogens, such as HIV-1, can easily target immune cells if recruited under these circumstances (Mikulak et al., 2017). Similar considerations may apply to the spread of MPOX in MSM. Though the infectious pustules associated with monkeypox can be disseminated through sexual contact, this does not mean the disease has been sexually transmitted.

## 4 Immunopathogenesis of MPOX

**Figure 3:**
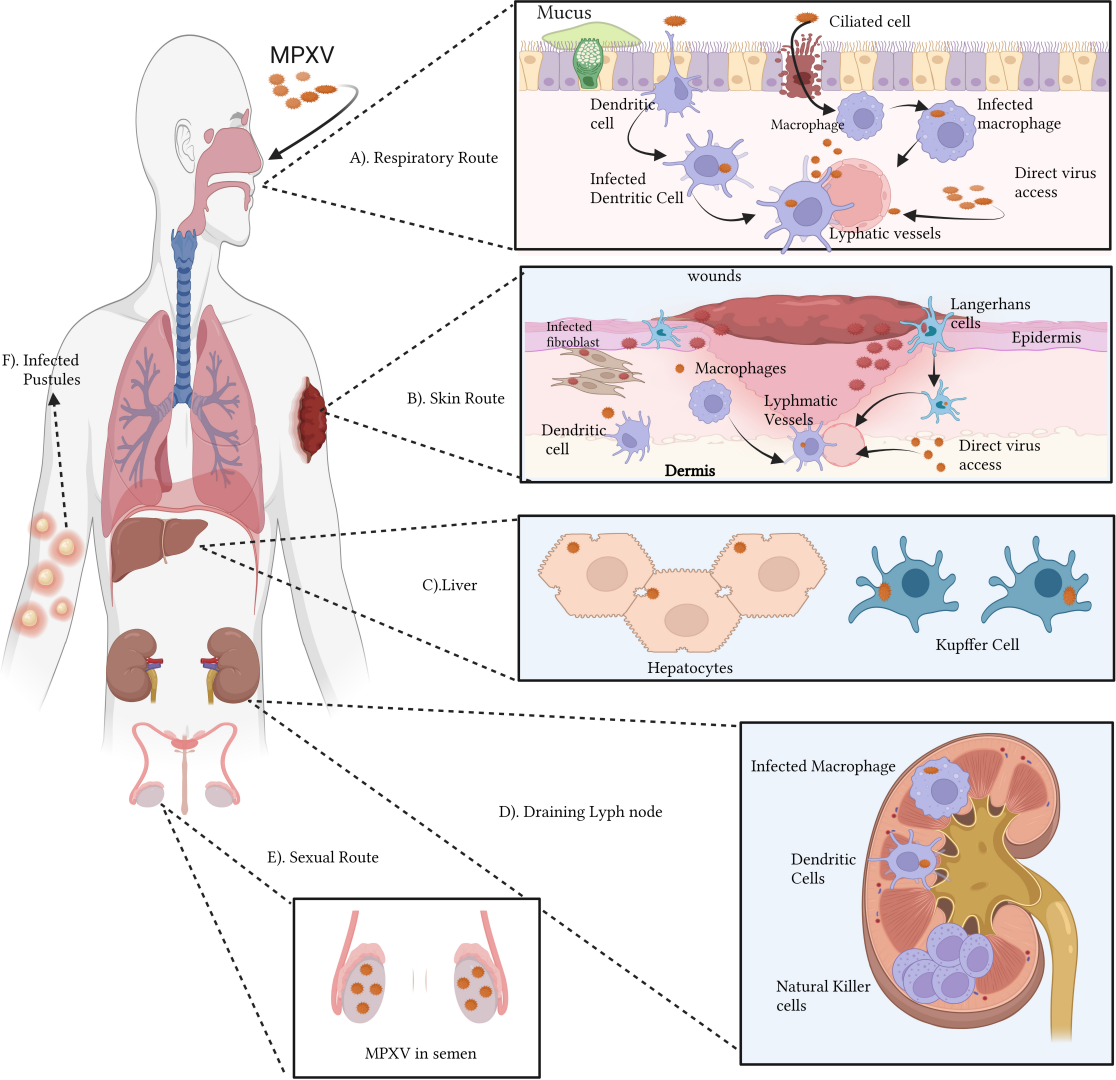
Immunopathogenesis of MPOX **(A–F)**: The monkeypox virus (MPOX) can infect a host through the respiratory **(A)** or cutaneous **(B)** pathways. Ciliated cells and airway epithelium are prone to respiratory virus infection. MPOX infects APCs dendritic cells and macrophages, Keratinocytes, fibroblasts, and Langerhans cells upon viral inoculation in the skin. Lymphadenopathy, a common feature of MPOX infection in the lymphatic system, is also hypothesized. The abnormal proliferation and retention of natural killer cells might be one of the causes. MPOX targets lymphoid tissue, then the spleen and liver. NHP hepatocytes and Kupffer cells contain MPOX antigens. **(C)**. Infected antigen-presenting cells may migrate to draining lymph nodes and contribute to lymphatic infection transmission **(D)**. Viraemia can transmit the virus to the skin and gonads. Semen with MPOX suggests sexual transmission **(E)**. Pustules result from infected skin and mucosa **(F)**. The figure is drawn by app.biorender.com.

Orthopoxviral infections in vertebrates can have a wide range of clinical manifestations depending on the virus’s entry route to establish infection (Stanford et al., 2007) (**Figure 3**). Aerosolized respiratory secretions or ingested bodily fluids from infected persons can spread various orthopoxviruses, including the extremely infectious VARV and MPOX, through the respiratory/oral cavity (Rimoin et al., 2010). The pathogen subsequently spreads to the mucosae of the mouth and lungs, infecting the epithelium lining the upper, middle, and lower airways (Zaucha et al., 2001). There are no symptoms of oropharyngeal lesions during this phase of infection. Antigen-presenting cells, including monocytes, macrophages, B cells, and dendritic cells (DCs), are situated close to infected cells, allowing the virus to infect and propagate to more tissues (Engelmayer et al., 1999; Humlová et al., 2002; Li et al., 2005; Stanford et al., 2007; Song et al., 2013b; Dai et al., 2014). Orthopoxviruses have been shown to spread to surrounding draining lymph nodes, but the exact methods by which this occurs are still up for debate. It has been shown, for instance, that DCs from infected mice move from the bronchial mucosa to the draining lymph nodes, a process that likely aids in viral transmission (Beauchamp et al., 2010). DCs generated from human monocytes have been demonstrated to be unable to sustain the early lymphatic transmission of VACV after infection, suggesting that DCs play no function in this phase of the virus’s life cycle. Importantly, within hours of inoculation, VACV has already spread to draining lymph nodes, suggesting that the virus uses lymphatic channels to spread (Engelmayer et al., 1999; Norbury et al., 2002; Liu et al., 2005; Walzer et al., 2005; Humrich et al., 2007; Hickman et al., 2013).

Orthopoxviruses proliferate extensively in lymphoid tissues after initially infecting draining lymph nodes. One of the signs of monkeypox is lymphadenopathy or inflammation of the lymph nodes. MPXV may attack additional significant organs like the spleen and liver after spreading through lymphatic tissue. Notably, nonhuman primate (NHP) models have revealed the presence of MPXV antigens in Kupffer cells and hepatocytes. During the viremic cycle, the virus may then travel to distant organs like the skin and gonads [through respiratory droplets, bodily fluids, contaminated items, and skin lesions—the crust of an infected person]. Although intimate physical contact is an established risk factor for transmission, it is unknown if sexual interaction specifically encourages the spread of monkeypox. Although liver involvement is unclear, there is evidence that lymphoid organs including the spleen and bone marrow promote VARV proliferation (Shafaati and Zandi, 2023). According to clinical research studies on human monkeypox, the throat and neck lymphatic tissues are sites of primary MPOX duplication (Song et al., 2013b). A model of aerosolized MPOX infection in cynomolgus macaques proved this notion, demonstrating that the mandibular, the tonsils, and mediastinal lymph nodes were sites of initial viral replication (Zaucha et al., 2001). Infection of activated T cells, B cells, DCs, and monocyte/macrophages has been linked to poxviruses in lymphoid tissue, suggesting that these cell types may also be MPOX’s targets (Chahroudi et al., 2005). Natural killer (NK) cells proliferated and increased in the lymph nodes at the site of inoculation in experimentally infected non-human primates (NHPs), but the processes leading to abnormal lymph node hypertrophy during spontaneous MPOX infection remain unclear (Song et al., 2013b).

Orthopoxviruses can spread throughout the body via the lymphohematogenous route after developing subclinical primary viremia due to lymphoid tissue infection (Zaucha et al., 2001; Chapman et al., 2010). After primary lymphatic spread, the spleen and liver are the principal organs infected in experimental mousepox models. After initial contagion in these areas, a subsequent large viremia wave is triggered, spreading the virus to other organs, such as the lungs, kidneys, intestines, skin, etc., through infected cells (Moulton et al., 2008). Viral antigen was detected in the liver of an NHP model of inhalational MPOX infection, with a heavy proportion in specialized macrophages like Kupffer cells (Zaucha et al., 2001). Hepatocytes, albeit to a lesser degree, also showed antigen detection. The human spleen and liver have been found to swell after MPOX infection (Pittman et al., 2022). The liver is also suspected of having a role in VARV infection, but there is less evidence for this than for the spleen and bone marrow (Council, 1999).

Orthopoxviruses initiate infection and skin lesion formation in the dermal tiny blood vessels (Council, 1999). However, it is unclear how the virus travels to the higher-stratified Skin, devoid of lymphatic and circulatory systems. Due to their susceptibility to VACV infection, migratory cutaneous DCs like Langerhans cells are likely culprits (Zaucha et al., 2001). Around the infectious pustule, CD3^+^ T cells, DCs, and macrophages have been shown to infiltrate (Alakunle et al., 2020). Despite cytotoxic T lymphocyte activation being associated with enhanced virus control in vaccinated rhesus macaques, further information is needed about the functionality of skin-infiltrating CD3+ T cells in MPOX infection (Edghill-Smith et al., 2005). An important aspect of MPOX infection is the development of epithelial lesions (enanthema) in the tongue, trachea, oropharyngeal mucosa, pharynx, larynx, and oesophagus, which can progress into ulcers that secrete infectious virus particles into the saliva (Council, 1999).

As an additional route of entry, skin infections are possible. In addition to infecting tissue-resident antigen-presenting cells, including DCs, Langerhans cells, macrophages, and monocytes, viruses can also employ mobile antigen-presenting cells to spread via the lymphatic system (Norbury et al., 2002; Deng et al., 2008; Cann et al., 2013). Despite this, recent research using an orthopoxvirus-infected mouse skin model suggests that VACV impedes DCs immigration from the skin to lymph node drainage (Aggio et al., 2021). Direct access to lymphatic vessels, as seen in Zika virus cutaneous infection models, may also play a role in the virus’s migration from the surface to the lymphatics (Reynoso et al., 2019).

### 4.1 Immune response of the human to MPOX

Despite the virus’s identification decades ago, human protection against MPOX infection has yet to be well described. As a result, it is common to extrapolate information on MPOX’s communication with the host defense system from research involving VACV and kindred orthopoxviruses. Here, we examine MPOX’s strategies to escape the immune system during an active infection and list the immune defenses the host may have to combat the virus.

#### 4.1.1 Host immune evasion by MPOX

**Figure 4:**
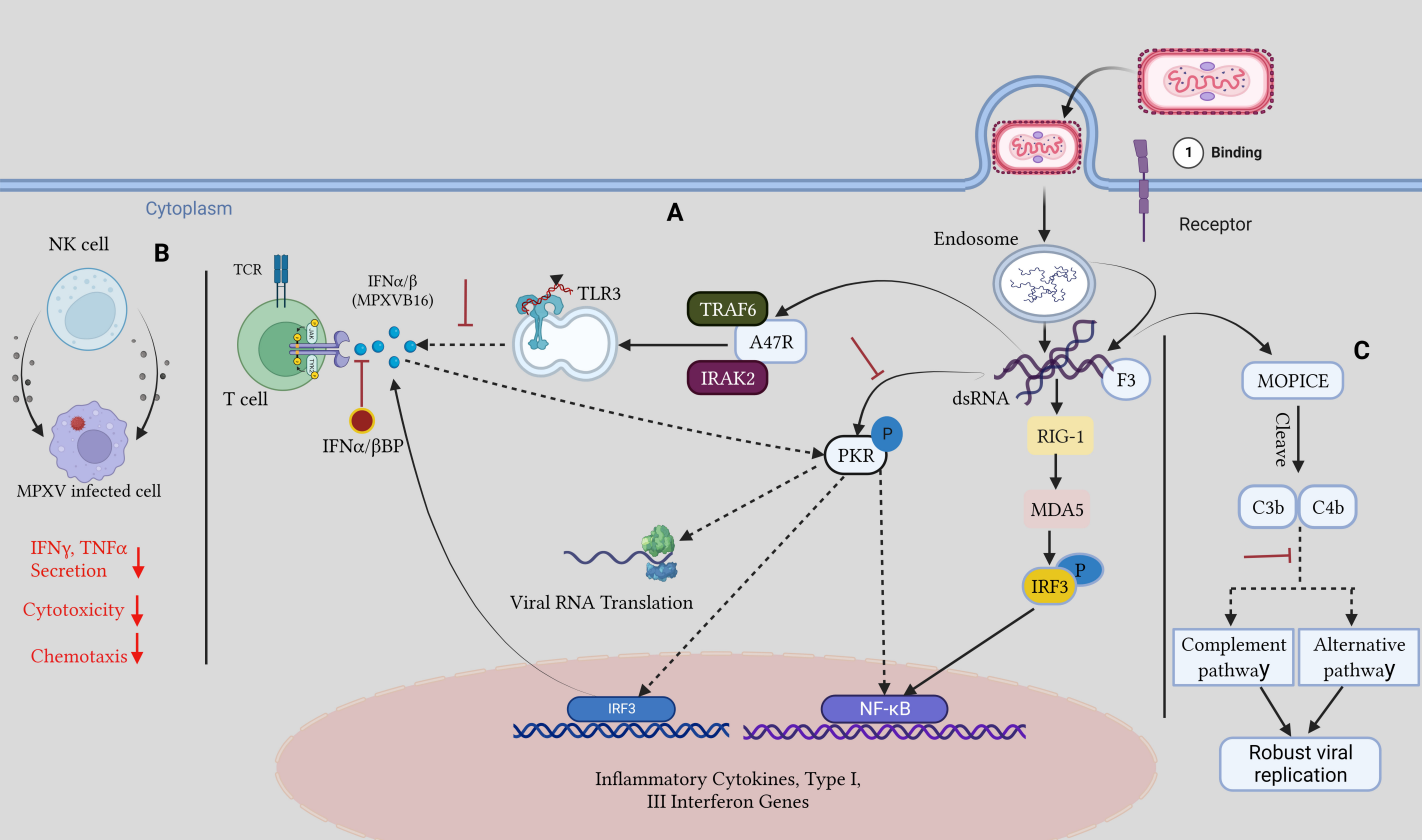
Host immune evasion mechanism by MPOX: **(A)** Upon viral entry, viral dsRNA intermediates are released, and the F3 protein attaches them and sequesters them away from PKR, inhibiting downstream antiviral pathways and Dampening the TLR3 response to them. IFN*α*/*β* BP hinders type I IFN binding to cells. **(B)**. After MPOX infection chemotaxis, IFN*γ* and TNF*α*, secretions and cytotoxicity are reduced in NK cells. **(C)**. The MOPICE protein hinders the host complement pathway, resulting in robust viral replication. The figure is designed by using app.biorender.com.

Orthopoxviruses are distinguished by their resistance to the host immune responses (Lu and Zhang, 2020; Yu et al., 2021). There are several virulence factors associated with monkeypox. Transcriptional analyses of infected cells have shown how MPOX infection avoids detection by TLR3. Even though there were no gene expression changes, cell death occurred after live MPOX infection of primary human fibroblasts and macrophages *in vitro*. In contrast, the interferon-sensitive genes are expressed during cellular infection with the MPOX virus, showing that the ability to evade the immune system is exclusive to the live virus. Reduced expression of interferon-responsive genes, IL-6, IL-1, IL-1, CCL5, and TNF-, was evidence that TLR3 signaling was downregulated during a live MPOX infection (Rubins et al., 2011; Smith et al., 2013; Nichols et al., 2017; Suraweera et al., 2020). Co-infected people rely heavily on this immune evasion strategy, as intact TLR3 signaling helps to regulate HIV replication by activating type I IFN and NF-kB signaling (Zhou et al., 2010). In addition, MPOX encodes the A47R protein, which binds to the cytoplasmic proteins TNF receptor-associated factor 6 (TRAF6) and interleukin-1 receptor-associated kinase 2 (IRAK2), which are required for TLR3-mediated activation of NF-*k*B (Bowie et al., 2000; Harte et al., 2003; Seet et al., 2003). The virus can avoid the Type I IFN response through several different methods. The E3L gene in VACV significantly reduces the cellular IFN-mediated antiviral immune response. The E3 protein can bind double-stranded RNA and secrete it from recognized pattern recognition receptors such as protein kinase R, MDA-5, RIG-I, and OAS to stop their activation. The two conserved domains of the VACV-E3 protein are the N-terminal Z-nucleic acid binding domain and the C-terminal dsRNA-binding domain (Zandi et al., 2023). Infected cells secrete a soluble IFN*α*/*β* binding protein called MPOXB16, which blocks Type I IFNs from binding to IFN receptors. IFN*α*/*β* binding proteins have been studied as a potential vaccine target because previous studies revealed that immunization against these proteins protects mice from lethal mousepox infection (Xu et al., 2008; de Marco M del et al., 2010). Type I and II IFNs are produced mostly by NK cells. MPOX inhibits chemokine receptor expression within the first week of infection, which is crucial for NK cell motility, cytotoxicity, and the release of TNF-*α* and IFN-γ, all of which contribute to the efficacy of NK cell-mediated clearance (Song et al., 2013b). MPOX also expresses the F3 protein, and it binds to dsRNA viral intermediates to block Protein kinase R (PKR) recognition of viral dsRNA (Arndt et al., 2015) (**Figure 4**).

MOPICE, an inhibitor of the monkeypox complement enzyme, suppresses the complement response of the host. There is evidence that MOPICE is present in the more dangerous Central African clade, but no evidence has been found in the West African clade, including the 2022 outbreak (Chen et al., 2005). Expression of MOPICE did not affect virulence in the West African clade, suggesting that it is not the primary determinant of differences in virulence between the two clades. Comparing MOPICE to proteins from the variola and vaccinia viruses allowed researchers to determine its role. These proteins cleave C4b and C3b to block the classical and alternative complement pathways (Liszewski et al., 2006; Hudson et al., 2012). Viral replication is boosted, and an improved adaptive immune response is largely owing to complement avoidance by the host. Elevated levels of anti-MPOX antibodies, greater viral loads, and increased T cell production of TNFγ- and IIFN-α were seen in MOPICE-deficient MPOX-infected rhesus macaques, which aid in infection clearance (Estep et al., 2011).

#### 4.1.2 Response of the innate immune system to MPOX

Innate immune cells are usually the first line of defense after an active viral infection, but some viruses can also attack these cells. Poxviruses have been shown to infect monocytes predominantly in many *in-vitro* and *in-vivo* experiments (Jahrling et al., 2004; Rubins et al., 2004; Hammarlund et al., 2008; Rubins et al., 2011; McCollum and Damon, 2014). It has been proposed that early identification of poxvirus antigens in neutrophils and monocytes is a reliable indicator of MPOX lethality. As a result of viral pneumonia brought on by MPOX infection, the number of CD14^+^ monocytes significantly augmented in cynomolgus macaques lungs (Johnson et al., 2011; Song et al., 2013a). Additionally, it has been demonstrated that mouse CD45^+^CD11b^+^GR-1^int^ inflammatory monocytes are tolerant to VACV replication and may function as plausible vectors for virus transmission. The replication and dissemination of VACV were also aided by human primary M2-like macrophages (Hickman et al., 2013; Byrd et al., 2014). Branching structures, lamellipodia, cell connections, and actin tails emerged in these primary macrophages in response to VACV infection, suggesting they may contribute to virus dissemination. Depletion of phagocytic cells, however, was also observed to not completely stop the spread of VACV in infected mice, indicating that other immune cells than monocytes and macrophages are also capable of promoting virus spread. However, neutrophils and Ly6G+ monocytes of the innate immune system invaded virus-infected cells and governed them, preventing viral tissue injury (Fischer et al., 2011; Hickman et al., 2013). The results were supported by inferential evidence from studies that correlated low blood neutrophil counts to fatigue in MPOX-infected mice (Nagata et al., 2014). Due to the limited ability of innate immune cells at the infection site to regulate the progression of pathogenesis and tissue pathology beyond the infected area, a systemic immune response is essential for stopping the spread of infection (Davies et al., 2017).

An integral part of innate immunity, NK cells can influence the adaptive immune response so that monocytes cannot (Paust et al., 2010). NK cell populations increase dramatically in the peripheral blood and lymph nodes of MPOX-infected rhesus macaques (by a mean factor of 23.1 times by 7 days post-infection and 46.1 times by 8-9 days post-infection, respectively). However, MPOX infection severely impeded the roaming capability of the major NK cell subsets, which negatively impacted their employment in the lymphatic and inflammatory tissues prior to this rapid expansion. It was also reported that chemokine receptors on these cells, including CXCR3, CCR5, CCR6, and CCR7, were down-regulated. NK cells have been shown to lose their degranulation and secretion of IFN and TNF after being isolated from lymph nodes and blood. However, in the CAST/EiJ mouse model, NK cell regulation of MPOX viral load was demonstrated, despite the lack of a correlation between NK cell count and viral clearance in this NHP model. This strain’s low number of NK cells makes it highly vulnerable to orthopoxvirus infection. CAST/EiJ mice were protected from mortality by IL-15 therapy when CD8^+^ and CD4^+^ T cells were eliminated prior to infection with MPOX (Song et al., 2013b; Earl et al., 2020). Considering that IL-15 therapy is known to enhance the amount of IFN-secreting NK cells transiently and CD8^+^ T cells, it is likely that the increased NK cells were accountable for the shielding effect (Jayaraman et al., 2014). NK cells help control ECTV (Ectromelia virus) and vaccinia virus (VACV) infections in C57BL/6 mice. Inducing mousepox in experimental animals, ECTV is a mouse-specific orthopoxvirus widely used to simulate other orthopoxviruses with therapeutic relevance (Bukowski et al., 1983; Fenner, 2000; Fang et al., 2008). CD94 expression on NK cells in C57BL/6 mice was hypothesized to be crucial for resistance to ECTV infection, which added an intriguing layer of complexity to the situation. The NK cell receptors NKG2E and CD94 orchestrate this process by binding Major histocompatibility (MHC) class I complexes containing the infected-cell-generated peptide Qa-1b. There is mounting evidence that NK cells can guard against ECTV infection through NKG2D, and it has been speculated that CD94-NKG2E and NKG2D may work synergistically to generate highly protective NK cells (Fang et al., 2008; Fang et al., 2011). Though, more research will be needed to fully comprehend the processes underlying this apparent synergism and the functions of NK cells in mousepox infections. Similarities between human and rat CD94 and NKG2 receptors imply they may offer protection against MPOX infection in humans (Vance et al., 1999). Neutrophils, macrophages/monocytes, NK cells, plasmacytoid DCs, conventional DCs, and innate lymphoid cells are unknown in MPOX-infected people. Understanding the functions of immune cells and identifying prognostic indicators during MPOX infection would require a thorough analysis and outline of these cells.

In animal studies, disease progression is correlated with the systemic cytokine responses triggered by VARV infection. Host gene expression was significantly altered in vaccinated cynomolgus macaques infected with VARV but not in uninfected macaques. In particular, MX1, MX2, IP10, OAS1, OAS2, OAS3, STAT1, STAT2, and PKR were enriched in many interferon-associated genes whose transcription remained induced by type II interferons. Two of the seven mice that succumbed to the sickness had impaired interferon responses, proving that a prompt response can save lives. The human IFN- inhibits the replication and dissemination of MPOX; MPOX does not robustly stimulate TNF-regulated genes and NF-B-regulated, exclusively in mice that surrender to the infection (Rubins et al., 2004; Johnston et al., 2012). The NF-*k*B and TNF pathways are affected by genes present in VARV and other orthopoxviruses (Shisler and Jin, 2004; Mohamed and McFadden, 2009; Shchelkunov, 2012; Suraweera et al., 2020).

Aberrant immunological signaling can impact infection outcomes despite the need for host immunity. Another study using VARV-infected cynomolgus macaques reported cytokines such as IFN, IL-6, CCL4 (also recognized as MIP1), CCL2 (also identified as MCP1), and IL-8 were all elevated in the first 4 days after infection. Due to the monocytosis induced by these cytokines, monocytic cell-associated viremia may become more widespread. The macaques died from VARV infection, which may have been brought on by a “cytokine storm” induced by an overwhelming number of these cytokines (Jahrling et al., 2004; Dutta and Nahrendorf, 2014). Furthermore, cynomolgus macaques infected with MPOX had higher levels of sCD40L, IFN, G-CSF, GM-CSF, CCL5 (also known as RANTES), CCL2, IL-2, IL-6, IL-8, and IL-1RA. In addition, CD14+ monocytes increased by a factor of 0.97–16.3% throughout critical infection, signifying that the overall immunological milieu encouraged the expansion and recruitment of monocytes in response to MPOX infection (Johnson et al., 2011).

Multiple cytokines, such as CCL5, CCL2, IL-17, IL-15, IL-13, IL-8, IL-6, IL-5, IL-4, IL-1, IL-2R, and IL-1RA, are documented to be upstretched in humans with MPOX infection after infection (independent of illness stringency). In patients with significant illness (>250 lesions), CCL5, GM-CSF, IL-10, and IL-2R concentrations were greater, but pro-inflammatory IL-6 levels were lesser. This cytokine profile shows an elevated amount of IL-13, IL-10, and IL-4, characteristic of a T helper 2 cell response. Diminished levels of IFN, TNF, and IL-2 display an anti-inflammatory milieu based on regulatory T cells (Johnston et al., 2015).

It has been shown that VACV can avoid immunological feedback by suppressing antiviral and inflammatory immune responses (van den Broek et al., 2000; Liu et al., 2005; Howell et al., 2006), and MPOX may employ a similar approach to destabilize host immunity (Thakur et al., 2019). To better understand MPOX pathophysiology and identify immunological correlates of protection, more research is needed to uncover the functional links between cytokine patterns and immune cells.

#### 4.1.3 Response of B-cells mediated humoral immune system to MPOX

Successfully eliminating smallpox through a worldwide immunization campaign with a live VACV vaccine proved the relevance of immunoglobulins and B cells in combating poxviruses for the first time (Cherry et al., 1977; Strassburg, 1982). After receiving VIG (vaccinia immune globulin) created from vaccine serum, people who come into close contact with smallpox patients are effectively protected from catching the disease (Kempe et al., 1961). VACV-specific B cell activation in rhesus macaques significantly protects against lethal MPOX infection (Edghill-Smith et al., 2005). Epidemiological Research shows that the VACV vaccine is effective against several poxviruses, not only MPOX (Jacobs et al., 2009). Amazingly, immunization produced VACV-specific memory B cells and antibody levels, and in some cases, these remained detectable more than 50 years after vaccination (Crotty et al., 2003; Bartlett, 2004). However, only around half of the vaccinated individuals >20 years post-vaccination had neutralizing antibody titers bigger than 1:32, the level at which protection from smallpox is believed to be achieved (Mack et al., 1972; Hammarlund et al., 2008). Cross-protective immunity against monkeypox is also expected to decline with time.

It has been established that human vaccines’ VACV-induced immunoglobulins cross-react with 14 MPOX proteins. Macaques infected with MPOX showed antibodies directed against three proteins (D8, H3, and A26) of the MPOX (Zaire-1979-005) genome (Keasey et al., 2010). Mature VACV virions adhere to surfaces via A26 and A27, which form a heterodimer to bind laminin. Additionally, orthologue H3 and D8 proteins are involved in this process (Lin et al., 2000; Chiu et al., 2007; Howard et al., 2008). Collecting IgM from freshly infected macaques revealed that these antibodies already knew to look for MPOX (Zaire-1979-005) proteins A33, A44, and C19, suggesting that these proteins may be exploited to develop antigen-based serological diagnostic tools (Keasey et al., 2010). Preventative therapy with a mixture of two mAbs (c8A and c7D11) protected marmosets from lethal MPOX infection, according to additional studies) (Mucker et al., 2018). c8A and c7D1 have recently been developed as possible mRNA vaccines encased in lipid nanoparticles; they target the VACV proteins L1 respectively (Wolffe et al., 1995; Chen et al., 2006; Mucker et al., 2022). Cross-neutralization against 4 clinically important orthopoxviruses, including live VARV and MPOX, was demonstrated using a combination of human-derived mAbs targeting the VACV proteins L1, B5, A33, A27, H3, and D8 (Gilchuk et al., 2016). However, MPOX-specific epitopes (linear and conformational) have not been widely described, even though we know the MPOX proteins identified by neutralizing antibodies.

Since IgM antibodies tend to predominate in initial immune reactions and IgG antibodies tend to predominate in subsequent immunological responses, it has been proposed that the antibody isotype distribution against the MPOX virus may be a key indicator of past immunity and protection. Among a cohort of 200 MPOX-infected patients recruited in the DRC between 2007 March and 2011 August, those with both IgM and IgG responses had a 5.09-fold higher likelihood of developing serious lesions compared to those with just IgG responses (Pittman et al., 2022). Patients with moderate or severe disease had higher anti-orthopoxvirus IgM titers than those with mild disease in a cohort of infected individuals from the 2003 MPOX epidemic in the United States, while anti-orthopoxvirus IgG responses were significantly abridged and less frequent in patients with moderate or severe disease (Karem et al., 2007). An initial response dominated by IgM, which is less efficient at avoiding disease, is likely to reflect low amounts of cross-protective IgG^+^ memory B cells. As a result, IgM responses could serve as a biomarker for the degree of illness. This also emphasizes the critical necessity to precisely characterize the antibody profile of people infected with MPOX across multiple cohorts. Similarly, the reasons why some vaccinated individuals develop secondary illnesses despite prior vaccination require investigation into the correlates of protection provided by the VACV vaccine.

#### 4.1.4 Response of T-cells mediated humoral immune system to MPOX

The CD4^+^ T cells, specifically T follicular helper cells, participate in memory B cell recall and maturation into antibody-secreting cells (MacLeod et al., 2009). Results showed that after immunization with VACV, memory CD4^+^ T cells had a half-life of 8-15 years and longevity of at least 50 years. The activation of these VACV-specific CD4^+^ T cells resulted in the production of IFN and TNF (Walzer et al., 2005). However, there was no reported link between anti-VACV antibody titers and the number of virus-specific CD4^+^ T cells (Hammarlund et al., 2003). Rhesus macaques immunized with VACV were demonstrated to mount a defensive antibody response against deadly MPOX infection, but this was found to be highly dependent on the amount of CD4^+^ T cells. Vaccinated macaques infected with SIV (Simian Immunodeficiency Virus) but with low CD4^+^ T cell numbers (300 cells/mm^3^) didn’t develop protective VACV-specific IgG and died after an MPOX challenge (Edghill-Smith et al., 2005). Low CD4^+^ T cell counts are observed in VACV- unvaccinated and vaccinated people with abandoned HIV-1 infection (Jayani and Sulistyawati, 2020). As a result, exposure to MPOX can cause serious illness in this population. Additionally, the virus may acquire mutations that increase its virulence or transmission capability if the patient has a more complex disease. A recent HIV-1 antiretroviral therapy patient infected with MPOX had a CD4^+^ T cell count of >700 cells/mm^3^ and did not exhibit severe illness (Smith et al., 2005; Sklenovska and Van Ranst, 2018; Hammerschlag et al., 2022). This data raises the possibility that CD4^+^ T lymphocytes play a crucial role in modulating the severity of monkeypox. However, further research is needed to completely comprehend the function of CD4^+^ T cells in MPOX contagion.

T cells can perform direct antiviral activities in addition to aiding antibody production. Since MPOX and other orthopoxviruses replicate within macrophages, cytolytic T cells can play a crucial role in clearing the body of infected macrophages and stopping further viral replication (Zaucha et al., 2001; Jahrling et al., 2004; Rubins et al., 2004; Hammarlund et al., 2008; Rubins et al., 2011). In a mouse model of VACV infection, CD8^+^ T cells were found to eliminate virus-infected monocytes and reduce viral spread (Hickman et al., 2013). For CD8^+^ T cells to be activated in response to VACV infection, T cells must present VACV peptides via MHC class I molecules (Dai et al., 2021). T cells also secrete IL-1 and IFN, which help activate CD8^+^ T cells by increasing the expression of the co-stimulatory molecules CD80 and CD86 (Dai et al., 2021). Mouse models of pulmonary infection with VACV have shown that IFN production by primary stimulated effector CD8^+^ T cells protects against mortality. IFN produced by CD8^+^ T cells alone was adequate for fortification, even in the absence of CD4^+^ T cells and B cells, suggesting that CD8^+^ T cells may also give a shield against infection with additional orthopoxviruses (Goulding et al., 2014). Memory CD8+ T cells, produced after VACV vaccination, have also been demonstrated to protect against lethal ECTV infection in mice. Increased immunity results from the protective actions of memory CD8^+^ T cells, which work in tandem with primary effector CD8^+^ T cells to produce IFN and perforin (Remakus et al., 2013). Moreover, after receiving the traditional smallpox vaccine by scarification, people’s immune systems were primed to produce cytotoxic CD8^+^ T cells and interferon-producing T cells (McClain et al., 1997; Ennis et al., 2002). This finding was corroborated by a different investigation when the subjects were given the live vaccinia smallpox vaccine. After vaccination, significant IFN fabricating CD8^+^ and CD4^+^ T cells were found (Miller et al., 2008). Genes involved in cytolytic activities were shown to be upregulated in activated CD4^+^ T cells from patients infected with VACV (Munier et al., 2016). Vaccinated individuals have also shown MHC class II-restricted cytolytic CD4^+^ T cells (Littaua et al., 1992). Virus removal in vaccines with deficient or absent memory CD8^+^ T cell responses may be attributable to these cells (Hammarlund et al., 2003). Researchers have found that perforin-dependent cytolytic CD4^+^ T cells are present in the mousepox model (Fang et al., 2012). These findings underscore T lymphocytes’ critical role in containing orthopoxvirus infections.

Multiple CD8^+^ and CD4^+^ T cell epitopes have been discovered across the orthopoxvirus proteome in humans, mice, and NHPs (Kennedy and Poland, 2007; Sette et al., 2009; Walsh et al., 2009). Many are conserved among the most significant orthopoxviruses and attach to class I and II MHC molecules (Calvo-Calle et al., 2007; Kennedy and Poland, 2010; Molero-Abraham et al., 2015). Specifically, CD8^+^ T lymphocytes precise for two discovered epitopes in the immediate-early E3 protein of VACV (class I-restricted MHC NPVTVINEY and class II-restricted MHC GRVFDKADGKSKRDA) were able to destroy infected cells and block the dissemination of VACV (Ando et al., 2020). Both epitopes are expressed in the MPOX analogue, represented by the MPOX F3L gene (Arndt et al., 2015). Previous studies in cynomolgus macaques exhibited that VACV lacking in E3 protein did not guard MPOX (Denzler et al., 2011). The detection of E3 protein during the first 30 minutes of a VACV infection should be easily digested and offered by infected cells, permitting T cell-mediated lysis before virion generation and release (Resch et al., 2007). The combination of these features makes E3 a promising option for use in developing vaccines against the major orthopoxviruses in the future.

Although T cells may play a role in disease immunity, immunization against smallpox does not always result in strong T cell-mediated immunity against MPOX. Orthopoxvirus-specific CD8^+^ and CD4^+^T lymphocytes were undetectable in 2 of 5 vaccinated people who later contracted MPOX. Orthopoxvirus-specific CD4+ T cell response was identical across vaccinated and unvaccinated people, although orthopoxvirus-specific CD8+ T cell responses were more robust in unvaccinated patients (Karem et al., 2007). There is yet no compelling evidence linking CD4+ and CD8+ T cell responses to the severity of MPOX infection in humans.

## 5 Diagnosis

The prompt prevention of monkeypox epidemics can be the aided by precise, sensitive, and speedy detection of MPOX. Antibody detection (IgG/IgM serology testing), a particular peptide-based rapid antigen test (RAT), and nucleic acid detection (Real Time-PCR, etc.) are all currently accessible methods for identifying human MPOX. However, the technologies currently available aren’t precise enough to distinguish MPOX infection from other orthopoxvirus infections. The recommended diagnosis of MPOX infection is the detection of viral DNA using either real-time or traditional PCR. In addition to species-specific probes/primers PCR, sequencing, and restriction length fragment polymorphism (RFLP) can also be employed alone. Some strategies require at least two stages: First, a PCR test can detect MPOX but cannot identify a specific subtype; next, PCR amplicons can be sequenced to detect the MPOX subtype specifically. The effective detection of MPOX DNA in clinical and veterinary specimens and MPOX-infected cells has been described using multiplex polymerase chain reaction (MPCR) targeting conserved genes such as E9L, complement binding protein (C3L), F3L, G2R, envelope protein gene (B6R), hemagglutinin (HA), and N3R (Kulesh et al., 2004; Li et al., 2006; MacNeil et al., 2009; Grant et al., 2010; Li et al., 2010; Maksyutov et al., 2016; Li et al., 2017; Yinka-Ogunleye et al., 2019). Although RFLP can reveal viral sequences, it is a time-consuming and costly process that calls for polyacrylamide gel electrophoresis, restriction enzyme digestion, and virus culture. Recently, RPA (recombinase polymerase amplification) test targeting the G2R gene has made rapid virus detection possible (as short as 7 minutes) (Davi et al., 2019). For the speedy and accurate detection of MPOX, scientists have created a LAMP (loop-mediated isothermal amplification) assay. The LAMP test can analyze MPOX infection and distinguish between MPOX strains found in West Africa and those found in the center of Africa (Iizuka et al., 2009). Incredibly, whole-genome next-generation sequencing (NGS) is now considered the gold standard for characterizing orthopoxviruses, particularly MPOX (Farlow et al., 2010; Cohen-Gihon et al., 2020). NGS simultaneously recognizes DNA bases while integrating them into a nucleic acid chain utilizing clonal amplification and sequencing by synthesis (SBS) chemistry to enable rapid and precise sequencing. The technology has great potential but comes at a high price in terms of money, time, and data processing. Thus, NGS might not be a good choice for characterization in places with limited resources, such as sub-Saharan Africa. In addition to the current standard of care in PCR, - based methods for detecting MPOX, real-time viral genome data is required for evidence-based epidemiological interventions, and this cannot be obtained without field genome sequencing equipment like the Nanopore MinION (Vandenbogaert et al., 2022). The primary function of the Minion is to perform real-time DNA base reading via Nanopore Sequencing. Minion nanopore sequencing operates on a very fundamental principle: electrophoretically driving DNA or RNA strands into a nanopore.

### 5.1 Treatment of MPOX

#### 5.1.1 Antiviral drugs of MPOX

Supportive care is the only treatment considered to be the standard of care for monkeypox at this time (Rizk et al., 2022; US, Centers for Disease Control and Prevention, 2022). A minority of people infected with MPOX may experience severe illness and require treatment. For those who do, antibiotics to combat secondary infections and systemic antiviral drugs are common options (Adler et al., 2022) (**Tables 1A**, **B**). Research into the efficacy of cidofovir, a smallpox vaccine given after exposure, and a related antiviral acyclic nucleoside phosphonate analogue in treating a fatal dosage of MPOX in monkeys was conducted in 2006 (Stittelaar et al., 2006). Despite the effectiveness of both antiviral drugs in reducing disease severity, smallpox immunization following exposure did not reduce mortality. The antiviral cidofovir is typically prescribed as a DNA polymerase inhibitor for treating CMV retinitis. Although the drug has shown some efficacy in treating MPOX in humans, its widespread application is constrained by the nephrotoxicity commonly linked with cidofovir (https://www.cdc.gov/poxvirus/monkeypox/clinicians/treatment.html#anchor_1655488137245; Johnston et al., 2015). A lipid conjugate of cidofovir, brincidofovir, has lower nephrotoxicity. Three patients who were given brincidofovir in a case series from 2022 developed transaminitis. The drug did not affect clinical outcomes or virus levels (Adler et al., 2022). Antiviral drugs like tecovirimat work by blocking a viral envelope protein called p37, thereby preventing the virus from escaping the cell. Under an Investigational New Drug procedure, tecovirimat, licensed by FDA to treat smallpox infections, is being tested in patients of all ages with severe instances of MPOX (Almehmadi et al., 2022).

**Table 1A:** Antiviral medications can be used to combat the Monkeypox virus.

Antiviral therapeutic agents	Route of Administration	Mechanism of action	Use of particular populations	Drug interactions	Dosages	Adverse incidents	Approval by FDA for MXPV
Brincidofovir	PO	Lipid conjugate of cidofovir; Viral DNA polymerase inhibitor	Given that brincicovir can increase blood bilirubin and transaminases, liver function tests should be performed before and periodically throughout treatment.	Due to increased exposure, Brincidofovir-related side effects may be exacerbated by 1B3 and OATP1B1 inhibitors. Use an alternative medication that isn't a 1B3 or OATP1B1 inhibitor.	Adults: Two weekly doses of 200 mg once a week, Paediatrics: Two weekly doses of 6 mg/kg are recommended for children under 10 kg; for children 10–48 kg, the recommended weekly dose is 4 mg/kg.	Transaminitis, abdominal pain, nausea, Diarrhea, vomiting,	No
Cidofovir	IV	Viral DNA polymerase inhibitor	Renal function-based dosage modifications are needed: creatinine > 1.5 mg/dL, urine protein ≥ 100 mg/dL. CrCl ≤ 55 mL/minute, or serum	Nephrotoxic agents, probenecid	5 mg/kg weekly for 2 weeks, then 5 mg/kg every other week	Metabolic acidosis, anterior uveitis, nephrotoxicity, decreased intraocular pressure, neutropenia, teratogenicity,	Yes
Tecovirimat	PO, IV	A key envelope protein, Stop viral release by blocking p37	PO: No dose adjustment for the kidneys or liver is required when given. IV: Patients who have significant renal impairment are not good candidates.	Midazolam: reduced its efficacy Repaglinide: hypoglycemia	Adults: For 14 days, 600 milligrams twice a day, Pediatrics: For 14 days, take 200 mg; thereafter, for two weeks, take 400 mg twice a day (25-40 kg) or 600mg (13-25 kg)	Infusion site reaction, headache, neutropenia, nausea, abdominal pain, vomiting	Yes
Vaccinia immune globulin	IV infusion	Provides passive immunity to orthopoxviruses		Maltose content: a rise in blood sugar can reduce the effectiveness of immunizations against live attenuated viruses and can cause hypoglycemia if it isn't managed. This could potentially impact the results of serological tests; revaccination	Initially, 6000 U/kg; A dose of 9000 U/kg may be explored if the patient does not respond to the starting dose. depending on the severity of symptoms and therapeutic efficacy.	Headache, nausea, dizziness, aseptic meningitis, hemolysis	Yes

Although there is a lack of research specific to children, the following treatments have been applied in both pediatric and adult populations.

**Table 1B:** Number of confirmed cases by country (highest to lowest) as of November 10, 2022.

Country	Confirmed
United States	24198
Brazil	7205
Spain	7083
France	3934
Germany	3590
England	3412
Peru	2251
Colombia	1653
Canada	1388
Mexico	1367
Netherlands	1221
Portugal	908
Italy	837
Chile	783
Belgium	757
Switzerland	503
Austria	304
Nigeria	277
Argentina	265
Israel	250
The Democratic Republic Of The Congo	195
Sweden	186
Denmark	183
Ireland	178
Poland	173
Puerto Rico	170
Bolivia	155
Australia	132
Ecuador	93
Scotland	93
Norway	90
Ghana	84
Hungary	77
Greece	72
Czech Republic	66
Luxembourg	55
Slovenia	46
Wales	46
Serbia	40
Romania	39
Northern Ireland	34
Finland	33
Malta	33
Dominican Republic	31
Croatia	29
Singapore	19
United Arab Emirates	16
Guatemala	15
Slovakia	14
Panama	13
Jamaica	13
Iceland	12
India	12
Lebanon	11
Estonia	11
Turkey	11
Central African Republic	8
Saudi Arabia	8
Thailand	8
Cameroon	7
Martinique	7
Bulgaria	6
Uruguay	6
Gibraltar	6
Sudan	6
Honduras	6
Lithuania	5
Cyprus	5
Qatar	5
Venezuela	5
South Africa	5
New Zealand	5
Latvia	5
Philippines	4
Costa Rica	4
Andorra	4
El Salvador	4
Japan	4
Curaçao	3
Morocco	3
Aruba	3
Monaco	3
Republic of Congo	3
Taiwan	3
Benin	3
Bosnia And Herzegovina	3
South Korea	2
Greenland	2
Guyana	2
Bahamas	2
South Sudan	2
Georgia	2
Russia	2
Liberia	2
Montenegro	2
Moldova	2
Cuba	2
Saint Martin (French part)	1
Indonesia	1
Barbados	1
Bermuda	1
Jordan	1
Hong Kong	1
Egypt	1
Guam	1
Iran	1
Guadeloupe	1
New Caledonia	1
China	1`
Paraguay	1

Serologic treatments have also been taken into consideration for treating MPOX. In a study that was conducted in 2016, it was shown that persons who were immune to orthopoxviruses developed antibodies that were specific for a range of epitopes that are shared by many varieties of orthopoxviruses. Of these antibodies, 54% were able to neutralize the virus (Gilchuk et al., 2016). In addition, many monoclonal antibodies worked better than a single one at neutralizing orthopoxviruses. Some vaccine-related side effects and smallpox infections were once treated with a vaccine-induced immune globulin or vaccinia immune globulin (Wittek, 2006). Clinical usage of this agent has been considered for treating patients with T-cell lymphopenia who are not candidates for a live MPOX vaccination (Thornhill et al., 2022). Current FDA approval only applies to treating Orthopoxvirus infections throughout an outbreak. Therefore, its usefulness in treating MPOX is unknown.

#### 5.1.2 Vaccines against MPOX

Vaccination is thought to be the most effective defense to avoid orthopoxvirus infections. The humoral and cellular immune responses to an orthopoxvirus infection provide cross-protection against other orthopoxviruses. Consequently, smallpox vaccines are considered adequate to manage MPOX outbreaks, and vaccination is predicted to be 85% successful (https://www.cdc.gov/poxvirus/monkeypox/clinicians/smallpox-vaccine.html#anchor_1545415175541). However, there is currently no data on real-world effectiveness.

After the success of the smallpox vaccine, vaccine science had a tremendous advancement, which has resulted in greatly improved safety and efficacy. As a result, three successive generations of improved smallpox vaccines were created (World Health Organization, 2022) (**Table 2**).

**Table 2:** Vaccinia vaccinations to prevent MPOX and expanded Access Investigational New Drug Protocol (EA-IND).

Vaccine	LC16m8	JYNNEOSTM/MVA-BN	ACAM2000	Vaccinia (Dryvax, Lister, Copenhagen)	
**Generation**	III generation	III generation	II generation	I generation	
**Age group**	Infants, children, and adults (all ages)	General adult population			
			Adults (18–64)	–	
**Dosage**	single Dose	Double Doses, given 4 weeks apart	Single Dose	Single Dose	
**Approved for Monkeypox treatment**	No	Yes (USA, UK, Canada)	Yes (USA, UK, Canada)	No	
** ** ** ** ** ** ** ** **Structure**	Minimally replicating vaccinia virus.	Nonreplicating vaccinia virus	Propagated in tissue cell culture and produced under good manufacturing practices (live, replication-competent virus)	Several different strains of vaccinia virus propagated in calf lymph (live, replication-competent virus)	
** ** **Presentations**	Freeze-dried Multidose vials	Liquid frozen or lyophilized (freeze-dried) Single-dose vials (Multidose vials possible)		Liquid frozen or lyophilized vials or ampoules	
			Freeze-dried Multidose vials		
**Delivery mode**	Scarification	Subcutaneous or intradermal	Scarification	Scarification	
**Injection Materials**	Bifurcated needle	Needle and syringe (subcutaneous administration)	Bifurcated needle	Bifurcated needle	
**vaccination-site skin lesion**	Yes	No	Yes	Yes	
**Serious adverse effects**	Eczema vaccinatum, vaccinia infection	None	retrogressive vaccinia, eczema vaccinatum, autoinoculation, myopericarditis/ myocarditis, Steven-Johnson syndrome	
** ** ** ** ** ** ** ** **Precautions & contraindications**	Immunodeficiency dermatitis or other skin barrier disorders	Antibiotics like ciprofloxacin and gentamycin might cause allergic reactions in certain people.	• Allergy to certain medications, such as polymyxin B or neomycin	
			•Pregnancy	
			•Infants <12 months	
			• Other skin barrier disorders or eczema	
			• Cardiac infarction	
			•Immunodeficiency	
			• Due to the possibility of ocular vaccinia from autoinoculation, corticosteroid eye drops are used.	

#### 5.1.3 Dryvax a live viral vaccine (1^st^ generation)

Since smallpox was not yet eradicated in the United States, scarification was the primary delivery method for Dryvax, a live vaccinia strain cultivated on calfskin (Nalca and Zumbrun, 2010). Studies in rhesus macaques demonstrate that a positive serologic response to vaccination is necessary to protect against a lethal intravenous challenge with MPOX (Edghill-Smith et al., 2005). Dryvax recipients have reported serious side effects like acute vaccinia syndrome (with symptoms like malaise, fever, myalgia, and headache), vaccine-associated myopericarditis or myocarditis, Stevens-Johnson syndrome, subsequent pyogenic infections at the site of vaccine delivery, disseminated vaccinia, post-vaccination encephalitis, and autoinoculation leading to vaccinia in a location other than the primary vaccination site. Extreme cases of fatality due to these issues are quite rare (https://biotech.law.lsu.edu/blaw/bt/smallpox/dryvax_label.htm; Al-Musa et al., 2022). The adverse impact profile of Dryvax prompted the development of a new live vaccine, ACAM2000, which has now replaced it (DTaP et al., 2015).

#### 5.1.4 ACAM2000, live viral vaccine (2^nd^ Generation replication vaccines)

In a serum-free environment, the ACAM2000 strain was cloned from the Dryvax parent strain by plaque purification. Researchers found that vaccinated monkeys showed no clinical indications of sickness after exposure to the potentially fatal MPOX virus. The antibody titers of vaccinated monkeys were on par with those generated by Dryvax (Marriott et al., 2008). This replication-competent vaccinia strain is now presented through an EA-IND (Expanded Access Investigational New Drug) application for the treatment and prevention of monkeypox. In 2007, the FDA approved it for use in the United States as a preventative measure against smallpox in high-risk populations (Centre for Diseases Control and Prevention, 2022a). The scarification method of percutaneous administration provides a single dose of this vaccination. ACAM2000 shares many of the same negative effects as Dryvax, such as itching and soreness at the inoculation site, lymphadenitis, and acute vaccinia syndrome. A black box warning is required due to rare but serious adverse events similar to those caused by Dryvax. There is an estimated 1 fatality per 1 million unvaccinated people and 1 death per 4 million vaccinated people (Organization WH, 2022).

The effectiveness of ACAM2000 and Dryvax was examined in two phase III clinical trials (https://www.fda.gov/vaccines-blood-biologics/vaccines/acam2000-smallpox-vaccine-questions-and-answers). The first study enrolled 780 people who had never had a vaccinia vaccine and randomly assigned them to receive ACAM2000 or Dryvax. Both vaccinations effectively induce an immune response when tested using cutaneous response measurements. 1242 persons who had previously received a smallpox vaccination participated in the second experiment, getting either ACAM2000 or Dryvax (Nalca and Zumbrun, 2010). Dryvax was more efficient, inducing high titers of neutralizing antibodies in this reimmunized group. These results indicate that the efficacy of Dryvax and ACAM2000 is comparable in vaccinia-naive individuals, but Dryvax is superior in revaccinating patients. Live viral vaccinations, such as Dryvax and ACAM2000, provide a risk of deadly, systemic infections and are therefore not recommended for people with impaired immune systems (https://biotech.law.lsu.edu/blaw/bt/smallpox/dryvax_label.htm; Vaccines WHO, 2022). Pregnant women, individuals with a history of eczema, and children younger than 18 years old are discouraged from receiving live vaccinia vaccines because of the potential for fetal transfer, dermatitis vaccinatum, or both. During scarification with live vaccinations, the virus can be passed on to the person’s close contacts who lack a functioning immune system, causing them to develop eczema vaccinatum (Vora et al., 2008). Other people who are more likely to experience negative side effects with ACAM2000 include those with a history of heart disease and those who are allergic to the drug’s polymyxin B sulfate and neomycin components. As many as 5.7% of every 1000 first immunizations are thought to have a risk of vaccine-associated myocarditis (Organization WH, 2022).

#### 5.1.5 The MVA-BN (modified vaccinia Ankara-Bavarian Nordic) vaccine (3^rd^ generation non-replication vaccine)

Live, attenuated vaccinia Ankara-Bavarian Nordic (MVA-BN) vaccine is not capable of replication in cells from mammals. Originally, this vaccination was employed in the fight to eradicate smallpox (Volz and Sutter, 2017). This attenuated vaccinia strain is safer than previous vaccinations because it cannot multiply. It also has a far more favorable side effect profile. Injection-site responses, headaches, muscle pain, and swollen lymph nodes are the most common adverse events. Due to the inclusion of the antibiotic gentamicin (0.163 g per dose) and ciprofloxacin (0.005 g per dose) in the MVA-BN vaccine, individuals with a history of allergy to these drugs may require further monitoring or counseling to assess the risks and benefits of vaccination. Researchers found no evidence of harm to developing fetuses in their animal studies (Centers for Disease Control and Prevention (U.S.), 2022).

MVA-BN is effective against MPOX in scientific studies. The immunological response to MVA-BN was found to be more robust and to produce more neutralizing antibodies in research in monkeys compared to Dryvax. Mild or asymptomatic sickness was seen in MVA-BN-vaccinated monkeys, with some acquiring temporary skin lesions. In contrast, huge skin lesions were seen in unvaccinated monkeys who were seriously ill or died (Earl et al., 2004). Another investigation in rhesus macaques found that a single dosage of MVA-BN established immunity to MPOX more quickly than Dryvax. This was due to the higher dose of MVA-BN that can be safely administered (Earl et al., 2008). Phase 3 clinical trial evaluated MVA-BN with ACAM2000 in 440 persons for efficacy (Pittman et al., 2019). The ACAM2000-associated significant cutaneous reaction was evaluated by challenging one group with ACAM2000 and the other with MVA-BN twice subcutaneously, four weeks apart. The peak serum-neutralizing antibody titers produced by MVA-BN and ACAM2000 were quite similar. In addition, after being inoculated with ACAM2000, subjects who had been immunized with MVA-BN showed no signs of disease, while those who had only received ACAM2000 had 76 mm^2^ lesions (95% confidence interval: 70-87 mm^2^). Furthermore, a small randomized controlled trial was conducted to assess the immunological response to MVA-BN immunization in 24 persons with a history of hematopoietic stem cell transplantation at least two years prior to study enrollment. Compared to the placebo group, those who received MVA-BN had significantly higher neutralizing antibodies and vaccinia-specific T-cell responses (Walsh et al., 2013). However, the lack of a transplant-free control group makes it problematic to draw solid conclusions from this study. The results of this study cannot be extrapolated to the immunocompromised community because all participants had a limited or no transplantation history, no active graft versus host disease, and had not used immunosuppressive medications within the past 30 days.

In 2019, the FDA authorized the use of MVA-BN, a combination of the Imvanex, Imvamune, and JYNNEOS vaccines, to prevent smallpox and monkeypox when given in two separate subcutaneous doses spaced over four weeks (Administration F and D, 2019). In 2022, JYNNEOS will be available for intradermal administration after being approved by Food and Drug Delivery. Studies indicated that a lower intradermal dose of MVA maintained a similar antibody response in amplitude and kinetics while decreasing the cutaneous response to a challenge with Dryvax; hence this was the dose chosen (Administration USF and D (2022); Seaman et al., 2010; Wilck et al., 2010). Two dosages of MVA were studied in a clinical trial in 2015; one group received the drug subcutaneously (n = 149), while the other received it intradermally (n = 146). Liquid subcutaneous and lyophilized intradermal had similar peak-neutralizing antibody titers. Intradermal delivery permits five times as many doses per vial, whereas the lyophilized form has a longer shelf life (Frey et al., 2015).

#### 5.1.6 LC16m8 vaccine (3^rd^ generation freeze-dried, live-attenuated vaccine)

Another vaccination using the vaccinia strain LC16m8 is now being studied for its effectiveness. Vaccinia strain LC16m8 is an attenuated replicating strain that produces less of the viral envelope outside of cells due to a shortened B5R membrane protein. In the 1970s, thousands of Japanese children received a single vaccine by scarification to protect them from smallpox (Kenner et al., 2006; Kenner et al., 2006). Except for mild ulcers at the inoculation site, all symptoms were avoided by the MPOX vaccine LC16m8 in a trial with cynomolgus monkeys after intranasal infection (Saijo et al., 2006). Neutralizing antibodies and T cell-specific responses against vaccinia, variola, and MPOX were induced by LC16m8, but at lower levels, in phase I/II clinical research comparing LC16m8 to Dryvax (Kennedy et al., 2011). To prevent MPOX, vaccination with LC16m8 has not yet been approved.

#### 5.1.7 Prophylaxis

The Centers for Disease Control and Prevention (CDC) in the United States have created a post-exposure prophylaxis plan to control the 2022 outbreak and will use vaccinations for pre-exposure prophylaxis, where they will be given to patients before they are exposed to MPOX. It is highly recommended that people exposed to MPOX get the JYNNEOS or ACAM2000 vaccine within four days. Post-exposure prophylaxis within 14 days of exposure to MPOX may be considered in high-risk individuals, such as those with a history of eczema or compromised immune (Centre for Diseases Control and Prevention, 2022b).

#### 5.1.8 DNA vaccines against MPOX

In addition to the conventional I, II, and III-generation vaccines, DNA vaccines have been investigated as an MPOX immunization approach that avoids the requirement for the live virus. After exposing rhesus macaques to a lethal dosage of MPOX virus, researchers found that feeding the animals plasmid DNA encoding four vaccinia proteins using a gene gun (which injects a concentrated amount of DNA using compressed helium) saved the animals from dying (Hughes et al., 2014). Due to the impracticality of human gene gun vaccination, a separate study investigated intramuscular immunization using plasmid DNA encoding four vaccinia proteins, both alone and in conjunction with the recombinant proteins. Injecting plasmid DNA into the muscle of rhesus macaques did not protect them from infection or death. The recombinant protein vaccine-treated macaques had a severe illness, but they ultimately recovered. Mild illness that cleared up in a few days was seen after vaccination with recombinant proteins, plasmid DNA, and protective antibody titers were produced for all four proteins (Heraud et al., 2006). There is a need for more research into the creation and evaluation of DNA viruses with the potential to inhibit MPOX.

## 6 Limitations

There are still many important concerns that need to be explored. To better understand the mechanisms of immunological resistance against MPOX, more research is needed into the peripheral and mucosal immune responses during human MPOX infection. It is unclear if smallpox VACV or MPOX immunization can trigger mucosal immunity. Since MPOX and other poxviruses can transmit through the aerosols, it will be important to describe the mucosal immune responses (McCollum and Damon, 2014). To better understand MPOX-related respiratory complications, it is essential to learn more about the characteristics of tissue-resident memory T cells and IgA during infection (Lin et al., 2000). As MPOX DNA has been found in semen, it is important to describe the preputial mucosal immunity (Antinori et al., 2022).

Another objective of vaccine evaluation is to define the immunological correlates of protection, particularly for newer vaccinations intended for pregnant women and children. Therefore, what additional factors (such as behavioral, geographical, dietary, medical, immunological, or genetic) are involved besides not getting vaccinated? The seriousness of respiratory viral infection in young children has recently been revealed to be related to the quality of their innate immune responses (Heinonen et al., 2020). Moreover, similar to what was shown for SARS-CoV-2 infections, sick children’s T-cell and B cell responses are often lower than those of adults (Cohen et al., 2021; Toh et al., 2022). Investigating the adaptive immune responses of MPOX-infected infants may shed light on why they have more severe diseases and less effective vaccines. Young children and pregnant women are among the most vulnerable populations; hence it is crucial to comprehend the possible risks of vaccination (Ježek et al., 1987; Fine et al., 1988; McCollum and Damon, 2014; Mbala et al., 2017; Pittman et al., 2022). Most existing treatments and vaccinations have not been subjected to adequate clinical testing in these groups. Due to the increased risk of adverse events, certain necessary vaccines and medications (including brincidofovir and ACAM2000) are not recommended or are even contraindicated for these people. However, to curb the current outbreak, the UK Health Security Agency has advocated that homosexual and bisexual males at a greater risk of exposure should get vaccinated (Ryan et al., 2008; UK Health Security Agency, 2022). However, characterization is also needed for other populations, such as the elderly, people taking long-term drugs, and those with underlying metabolic illnesses whose manifestations may vary. Developmental information after congenital MPOX infection in the fetus is still lacking. Longitudinal surveillance of MPOX patients would also allow us to learn whether or not MPOX infection can have lasting consequences, as was seen following SARS-CoV-2 infection during the current epidemic. Healthcare organizations around the world need to work together to find ways to stop the spread of monkeypox. Priority should be given to containment operations, emphasizing detecting and isolating cases, tracking potential carriers, and vaccinating those at risk. This study is limited to describing the detail context of the recent outbreak of MPOX and disease immunobiology. However detailed discussion and extensive research is required to study the host immune interactions and pathogenesis associated with the disease.

## 7 Conclusion

Monkeypox can be contained through strict contact tracking and immunotherapeutic and preventative measures. Similarly, diagnostics based on serology can help track potential exposure sources and gain insight into a patient’s past. Considering the prevalence of vaccine-induced poxvirus immunity, any serological diagnostic methods must be MPOX-specific (Dubois et al., 2012). Clinically and epidemiologically, the expansion of MPXV is significant. MPXV and the vaccinia and variola viruses share antigens. There is a considerable serological cross-reactivity between them as a result. In non-invasive infections in monkeys, seroconversion has been identified. As a result, MPXV-induced silent disorders may be more widespread than previously thought. Given the virus’s rapid evolution, health management in areas that are not yet infected should be vigilant and actively get ready for a quick response when suspected or confirmed cases in people are seen.

## Data availability statement

The datasets used and analyzed during the current study are available from the corresponding author upon reasonable request.

## Author contributions

CZ, KW, and MQ designed the manuscript. CZ and KW supervised the study. MQ, XC, MT, UA, MS, SQ, SL, JM, GW, MF, and HS wrote the preliminary draft manuscript. MQ and XC, illustrated the figures. CZ and KW reviewed the preliminary draft manuscript. MQ, XC, MS, and SQ edited and finalized the manuscript. All authors contributed to the article and approved the submitted version.
