# Corrigendum: The prospective outcome of the Monkeypox outbreak in 2022 and characterization of monkeypox disease immunobiology

**DOI:** 10.3389/fcimb.2023.1284014

**Published:** 2023-09-18

**Authors:** Muhammad Suhaib Qudus, Xianghua Cui, Mingfu Tian, Uzair Afaq, Muhammad Sajid, Sonia Qureshi, Siyu Liu, June Ma, Guolei Wang, Muhammad Faraz, Haleema Sadia, Kailang Wu, Chengliang Zhu

**Affiliations:** ^1^Department of Clinical Laboratory, Institute of Translational Medicine, Renmin Hospital of Wuhan University, Wuhan, Hubei, China; ^2^State Key Laboratory of Virology, College of Life Sciences, Wuhan University, Wuhan, China; ^3^RNA Therapeutics Institute, Chan Medical School, University of Massachusetts Worcester, Worcester, MA, United States; ^4^Krembil Research Institute, University of Health Network, Toronto, ON, Canada; ^5^Department of Pharmacy, University of Peshawar, Peshawar, Pakistan; ^6^Department of Microbiology, Quaid-I- Azam University, Islamabad, Pakistan; ^7^Department of Biotechnology, Baluchistan University of Information Technology, Engineering and Management Sciences (BUITEMS), Quetta, Pakistan

**Keywords:** monkeypox, MPOX infection, MPOX vaccination, MPOX immunity, MPOX diagnosis

In the published article, there was an error regarding the affiliations for authors Siyu Liu, Kailang Wu and Chenglian Zhu. Author Siyu Liu should instead of having affiliation 1, have “State Key Laboratory of Virology, College of Life Sciences, Wuhan University, Wuhan, China”. Author Kailang Wu should instead of affiliation 1, have “State Key Laboratory of Virology, College of Life Sciences, Wuhan University, Wuhan, China”. Author Chengliang Zhu should, instead of affiliation 2 have “Department of Clinical Laboratory, Institute of Translational Medicine, Renmin Hospital of Wuhan University, Wuhan, Hubei, China”. The corrected affiliations appear above.

The authors apologize for this error and state that this does not change the scientific conclusions of the article in any way. The original article has been updated.

